# Visual detection of cortical breaks in hand joints: reliability and validity of high-resolution peripheral quantitative CT compared to microCT

**DOI:** 10.1186/s12891-016-1148-y

**Published:** 2016-07-11

**Authors:** A. Scharmga, M. Peters, A. van Tubergen, J. van den Bergh, J. de Jong, D. Loeffen, B. van Rietbergen, R. Weijers, P. Geusens

**Affiliations:** Department of Medicine, division of Rheumatology, Maastricht University Medical Centre, P.O. Box 5800, NL-6202 AZ Maastricht, The Netherlands; NUTRIM School of Nutrition and Translational Research in Metabolism, Maastricht University, Maastricht, The Netherlands; CAPHRI School for Public Health and Primary Care, Maastricht University, Maastricht, The Netherlands; Department of Internal Medicine, Viecuri Medical Center, Venlo, The Netherlands; Faculty of Medicine and Life Sciences, Hasselt University, Hasselt, Belgium; Department of Radiology, Maastricht University Medical Centre, Maastricht, The Netherlands; Department of Biomedical Engineering, Eindhoven University of Technology, Eindhoven, The Netherlands

**Keywords:** Imaging, Computed tomography, Hand, Bone, Rheumatoid arthritis

## Abstract

**Background:**

To study the reliability and validity of high-resolution peripheral quantitative CT (HR-pQCT) with microCT (μCT) as gold standard in the visual detection of cortical breaks in metacarpophalangeal (MCP) and proximal interphalangeal (PIP) joints.

**Methods:**

Ten cadaveric fingers (10 MCP and 9 PIP joints) were imaged by HR-pQCT and μCT and visually analyzed by two independent readers. Intra- and interreader reliability were evaluated for the presence (yes/no, kappa statistics) and the total number (intraclass correlation coefficient, ICC) of cortical breaks. Sensitivity, specificity, positive and negative predictive value (PPV respectively NPV) of HR-pQCT in detecting cortical breaks were calculated.

**Results:**

With HR-pQCT, mean 149 cortical breaks were identified and with μCT mean 129 (*p* < 0.05). Intrareader reliability for the presence of a cortical break per quadrant was 0.52 (95 % CI 0.48–0.56) and 0.71 (95 % CI 0.67–0.75) for HR-pQCT and μCT, respectively, and for the total number of cortical breaks 0.61 (95 % CI 0.49–0.70) and 0.75 (95 % CI 0.68–0.82). Interreader reliability for the presence of a cortical break per quadrant was 0.37 (95 % CI 0.33–0.41) and 0.45 (95 % CI 0.41–0.49) for HR-pQCT and μCT, respectively, and for the number of cortical breaks 0.55 (95 % CI 0.43–0.65) and 0.54 (95 % CI 0.35–0.67). Sensitivity, specificity, PPV and NPV of HR-pQCT were 81.6, 64.0, 81.6, and 64 % respectively.

**Conclusion:**

Cortical breaks were commonly visualized in MCP and PIP joints with HR-pQCT and μCT. Reliability of both HR-pQCT and μCT was fair to moderate. HR-pQCT was highly sensitive to detect cortical breaks with μCT as gold standard.

**Electronic supplementary material:**

The online version of this article (doi:10.1186/s12891-016-1148-y) contains supplementary material, which is available to authorized users.

## Background

Peri-articular cortical breaks are one of the characteristic features of bone involvement in rheumatoid arthritis (RA) and predictors of further radiographic progression [1, 2]. Early detection of cortical breaks is an important indicator for intensifying treatment in order to modify the disease course [3]. In daily clinic, conventional radiographs (CR) are considered the gold standard for detection of cortical breaks in the hand joints in rheumatic diseases. CR is widely available, fast to perform, relatively cheap, and extensively validated, however its sensitivity to detect structural bone changes is low compared to computed tomography (CT), MRI and ultrasound [4–7]. A novel, sensitive imaging technique is High-Resolution peripheral Quantitative Computed Tomography (HR-pQCT) [8]. HR-pQCT allows analysis of the cortical and trabecular microarchitecture of peripheral bones with an isotropic resolution of 82 micrometer (μm). This technique is now also applied for 3D assessment of the bone microarchitecture in the hand joints [8–10]. A study by Stach et al. has demonstrated that HR-pQCT is more sensitive than CR in detecting cortical breaks in the hand joints in RA and also in healthy controls [8]. However, the resolution of the HR-pQCT images can be of the same order as the thickness of the cortical bone in finger joints. Due to partial volume effects, it is possible that thin cortices are falsely identified as breaks. Therefore, in particular with thin cortices, the reliability, sensitivity and specificity of the measurements might be impaired and depend on the reader’s perception.

The aims of this study were 1). to investigate the intra- and interreader reliability, and 2). to determine the sensitivity, specificity, positive predictive value (PPV) and negative predictive value (NPV) of HR-pQCT in detecting cortical breaks in hand joints, using μCT images with a much higher resolution (18 μm) as gold standard. We were particularly interested in the methodology of identifying cortical breaks by HR-pQCT, not to study the clinical value of these cortical breaks. We hypothesized that HR-pQCT is a reliable and sensitive imaging method for identifying cortical breaks in hand joints compared to μCT.

## Methods

### Specimens

For this study, we used cadaveric specimen, because μCT imaging can only be executed in-vitro*.* MCP and PIP joints of ten female right hand human cadaveric index fingers were imaged by both HR-pQCT and μCT. The donors had dedicated their body by testament signed during life to the Department of Anatomy and Embryology of the University of Amsterdam, the Netherlands. The fingers were fixated in formalin.

### HR-pQCT and μCT image acquisition

HR-pQCT (XtremeCT1, Scanco Medical AG, Switzerland) scans were performed at clinical in vivo settings, ie at 60 kVp tube voltage, 900 μA tube current, 100 ms integration time and 82 μm voxel size. μCT (μCT 80, Scanco Medical AG, Switzerland) scans were performed at 70 kVp tube voltage, 114 μA tube current, 300 ms integration time and 18 μm voxel size. On HR-pQCT, the region of interest of the MCP joint covered an area of 18.04 mm, 220 slices and for the PIP joint 9.02 mm, 110 slices.

On μCT, the region of interest covered an area of 15.26 mm; 848 slices and for the PIP joint 9.45 mm, 525 slices (Additional file 1: Figure S1) (see supplementary data available at *BMC Musculoskeletal Disorders* online).

### HR-pQCT and μCT image analysis

Scans of HR-pQCT and μCT were exported in Digital Imaging and Communications in Medicine (DICOM) format and analyzed using Osirix (v.5.8.5 64-bit) multiplanar DICOM viewer. Differences in the extent of the scanned areas as well as in joint angles were noticed because the fingers were scanned horizontally on HR-pQCT and vertically on μCT. Corresponding first and last slices of the overlapping region were visually determined to ensure that the same region of interest was used in the detection of cortical breaks on both imaging modalities.

Two trained readers (AS and MP) independently scored the HR-pQCT and μCT images visually for the presence of cortical breaks. The readers received extensive training from Study grouP for xtrEme Computed Tomography in Rheumatoid Arthritis (SPECTRA) and have additional reading experience, before the current dataset was read. Readers were aware of the hypothesis of this study. The images were not anonymized. HR-pQCT images were first and independently scored from μCT images, with at least one day in between, and a two week interval for the rescoring by Reader 1.

A cortical break was defined as a clear disruption of the cortex, seen on two consecutive slices on two orthogonal planes (on transverse and on sagittal or coronal plane) on HR-pQCT, and similarly, but on nine consecutive slices on μCT to cover the same area as evaluated by HR-pQCT (Additional file 2: Figure S2).

To assess the location of the breaks in each joint, the transverse plane was divided into four quadrants: palmar, ulnar, dorsal and radial (Additional file 3: Figure S3). The phalangeal base and metacarpal head of the MCP joints, and proximal phalanx and distal phalanx of the PIP joints were separately assessed. In total eight quadrants per joint were analyzed, four in the proximal bone, and four in the distal bone of the same joint. Each joint was systematically analyzed per quadrant. Quadrants with large discrepancies between the readers (ie more than four breaks difference) were re-examined to identify reasons for discrepancy. Also, total volumetric bone mineral density (vBMD) of the specimens was calculated using HR-pQCT.

### Statistics

Descriptive analyses were done to calculate the total number of cortical breaks scored by the readers per quadrant for each imaging modality.

The difference in the total number of cortical breaks detected with HR-pQCT versus μCT was tested for statistical significance with Wilcoxon signed-rank test. Intra- and interreader reliability were calculated using Cohen’s Kappa (k) and intraclass correlations coefficient (ICC) with a two-way random model and absolute agreement. k value was calculated for the presence (yes/no) of a cortical break per quadrant and ICC values were calculated for the total number of cortical breaks per quadrant. k and ICC were calculated on the level of all available quadrants. Kappa values were also re-calculated, corrected for potential prevalence and bias within the kappa value (Prevalence-Adjusted Bias Adjusted Kappa, PABAK) [11, 12]. Reliability was rated according to Landis et al.: <0.00 poor, 0.00–0.20 slight, 0.21–0.40 fair, 0.41–0.60 moderate, 0.61–0.80 substantial, 0.81–1.00 almost perfect [13]. Sensitivity, specificity, PPV, and NPV of HR-pQCT in the detection of cortical breaks were calculated with μCT as gold standard. The mean value of the two readings of reader 1 (AS) was used for this purpose. Statistical analyses were performed with SPSS Statistics for Windows version 23.0 (IBM Corp., Armonk, NY).

## Results

The mean age of the donors was 85.1 ± 9.6 years, the medical history was unknown. Average vBMD was 203 mgHA/cm^3^ for MCP joints, 293 mgHA/cm^3^ for PIP joints and 245 mgHA/cm^3^ for the total joints. The scans of ten MCP and nine PIP joints with in total 152 quadrants were available for analysis. One PIP joint could not be evaluated due to a missing μCT scan. Furthermore, Reader 1 considered the quality of the images of the metacarpal head in one MCP joint as too low on μCT due to a protocol error during scanning. Therefore four quadrants were excluded in the analyses of the μCT images.

Table 1 shows the total number of cortical breaks each reader found on HR-pQCT and μCT images. The differences in scores between the first and second reading of Reader 1 were not statistically significant on HR-pQCT (139 vs 118 breaks, *p* = .064) and μCT (142 vs 156 breaks, *p* = .163). However, the difference in the mean score between HR-pQCT versus μCT was statistically significant (respectively, 129 and 149 breaks, *p* < 0.05). The total number of cortical breaks on HR-pQCT scored by Reader 1 (first reading) versus Reader 2 was not statistically significant (respectively, 139 versus 151 breaks, *p* = .288). On μCT, Reader 2 found significantly more breaks than Reader 1 (first reading) (241 vs 142 breaks, *p* < .001). In total 4 quadrants with large discrepancies between the readers were re-examined. Several reasons for discrepancy were identified:

**Table 1:** Number of cortical breaks scored per imaging modality

	HR-pQCT	μCT	*p* value
Reader 1 (first reading)	139 (7.3 ± 4.1)	142 (7.4 ± 4.0)	*p* = .562
Reader 1 (second reading)	118 (6.2 ± 2.6)	156 (8.2 ± 3.6)	*p* < .000
Mean score Reader 1	129 (6.7 ± 3.0)	149 (7.8 ± 3.6)	*p* = .018
Reader 2	151 (7.9 ± 3.3)	241 (12.6 ± 6.3)	*p* < .000

Total number of detected cortical breaks on the HR-pQCT and μCT scans (mean ± SD per joint)

*n* = 19 joints

Abbreviations: *HR-pQCT* high resolution peripheral quantitative computed tomography, *μCT* microCT

- On μCT we defined a cortical break when present on nine consecutive slices. Sometimes Reader 1 observed the cortical break on eight consecutive slices, hence not considering it a break, whereas Reader 2 observed it on nine consecutive slices, thereby fulfilling the criteria for a break.
- The smaller the break, the less agreement between the readers. An example of this discrepancy is shown in Additional file 4: Figure S4 panel A and B.
- Due to the low bone mineral density and thin cortices, there was low contrast in some cases (example in Additional file 4: Figure S4 panel C).
- Reader 1 considered a break as one large break, whereas Reader 2 counted several small cortical breaks inside the same large break (example in Additional file 4: Figure S4 panel D).

Table 2 shows the intra- and interreader reliability based on the 152 quadrants on HR-pQCT and 148 quadrants on μCT that were evaluated. Intrareader reliability was moderate to substantial for the presence of breaks (HR-pQCT: k = 0.52 and μCT: k = 0.71) and for the number of breaks (HR-pQCT: ICC = 0.61 and μCT: ICC = 0.75).

**Table 2:** Intra- and interreader reliability per imaging modality

	Intrareader (reader 1)			Interreader (reader 1 first reading versus reader 2)		
	k (95 % CI)	PABAK (95 % CI)	ICC (95 % CI)	k (95 % CI)	PABAK (95 % CI)	ICC (95 % CI)
HR-pQCT	.52 (0.48 to 0.56)	.53 (0.39 to 0.66)	0.61 (0.49 to 0.70)	.37 (0.33 to 0.41)	.38 (0.23 to 0.53)	0.55 (0.43 to 0.65)
μCT	.71 (0.67 to 0.75)	.72 (0.60 to 0.83)	0.75 (0.68 to 0.82)	.45 (0.41 to 0.49)	.47 (0.33 to 0.61)	0.54 (0.35 to 0.67)

Intra- and interreader reliability of HR-pQCT and μCT images based on 152 quadrants for HR-pQCT and 148 quadrants on μCT. Kappa value was calculated on the presence of a cortical break (yes/no) and ICC was calculated on the total number of cortical breaks in all quadrants

Abbreviations: *PABAK* prevalence-adjusted bias-adjusted kappa values, *ICC* intraclass correlation, *HR-pQCT* high resolution peripheral quantitative computed tomography, *μCT* microCT

Interreader reliability was fair to moderate for the presence of breaks (HR-pQCT: k = 0.37 and μCT: k = 0.45,) and for the number of breaks (HR-pQCT: ICC = 0.55 and μCT: ICC = 0.54). The values of PABAK were comparable (Table 2).

Sensitivity, specificity, PPV and NPV of HR-pQCT in the detection of cortical breaks with μCT as gold standard were calculated with the mean scores of Reader 1 (reading 1 and 2) and the score of Reader 2. The sensitivity was 81.6 %, specificity 64 %, PPV 81.6 %, and NPV 64 % respectively for Reader 1, and sensitivity 68.9 %, specificity 69.4 %, PPV 82.6 % and NPV 51.5 % for Reader 2 (Table 3 and Additional file 5).

**Table 3:** Sensitivity, specificity, positive predictive value and negative predictive value of HR-pQCT

Joints	Total number of breaks on HR-pQCT	Total number of breaks on μCT	Sensitivity	Specificity	Positive predictive value	Negative predictive value
MCP reader 1	68	79	82.1 %	65 %	86.8 %	56.5 %
MCP reader 2	83	156	65.6 %	62.5 %	87.5 %	31.3 %
PIP reader 1	61	70	81 %	63.3 %	75.6 %	70.4 %
PIP reader 2	68	85	75.6 %	77.4 %	81.6 %	70.6 %
All reader 1	129	149	81.6 %	64 %	81.6 %	64 %
All reader 2	151	241	68.9 %	69.4 %	82.6 %	51.5 %

Sensitivity, specificity, positive predictive value and negative predictive value of HR-pQCT in the detection of cortical breaks with μCT as gold standard based on the mean score of Reader 1 (readings 1 and 2), and the score of Reader 2

Abbreviations: *HR-pQCT* high resolution peripheral quantitative computed tomography, *μCT* microCT

**Fig. 1:**
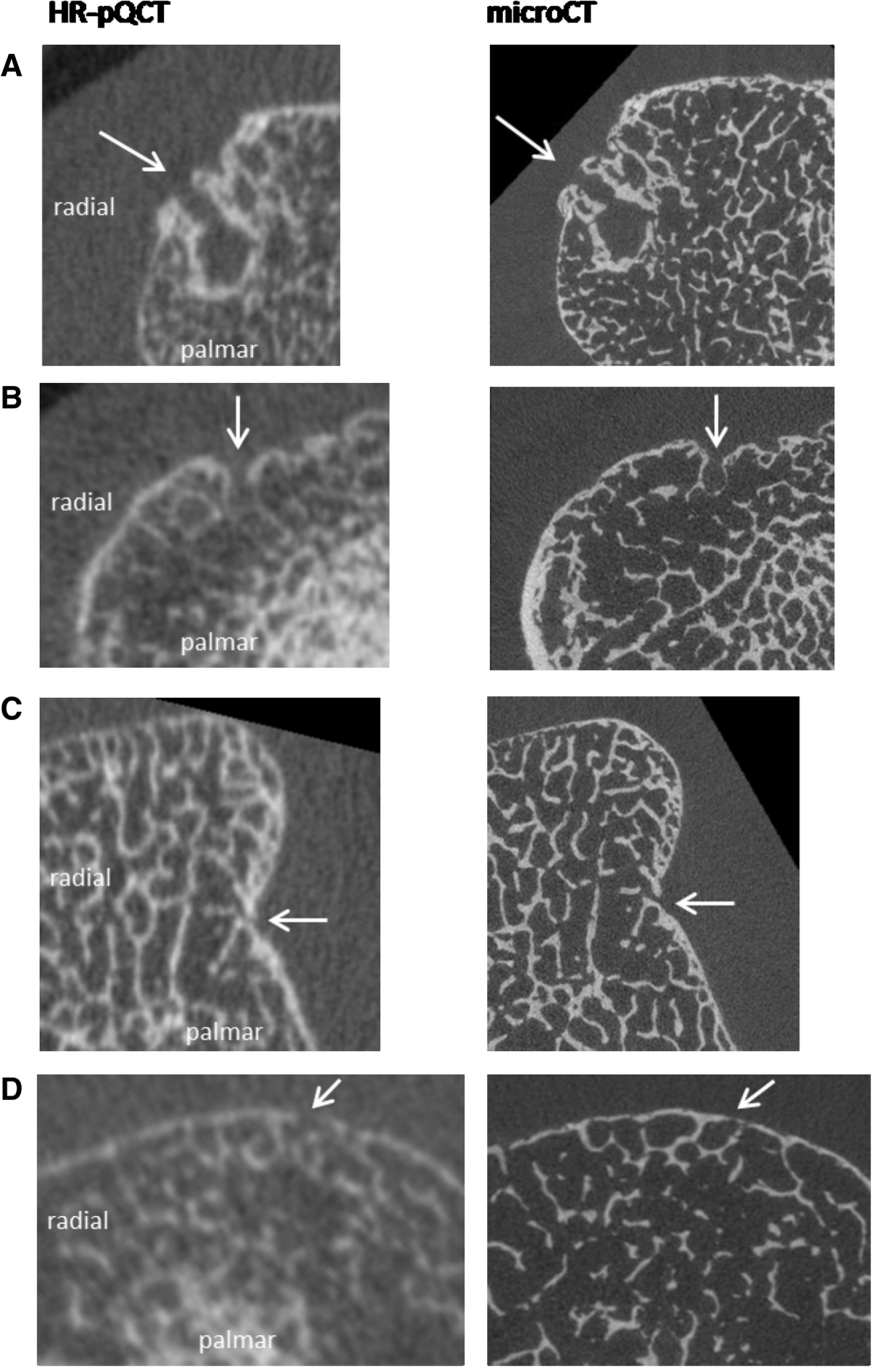
Corresponding images of cortical breaks on HR-pQCT and μCT. Panel **a** A discontinuity of the cortex (*arrow*) meeting the definition of a cortical break was seen on both HR-pQCT and μCT. Panel **b** A discontinuity of the cortex (*arrow*) meeting the definition of a cortical break was seen on both HR-pQCT and μCT. Panel **c** A discontinuity of the cortex (*arrow*) is seen on one slice only on HR- pQCT, thereby not fulfilling the definition for a break on HR-pQCT. A clear cortical break (*arrow*) is seen on μCT, which was also seen on nine subsequent slices, thereby fulfilling the definition of a break. Panel **d** A discontinuity of the cortex (*arrow*) meeting the definition of a cortical break is seen on HR-pQCT. On μCT, the cortical lining is intact (*arrow*). Abbreviations: MCP; metacarpophalangeal, HR-pQCT; high-resolution peripheral quantitative computed tomography, μCT; microCT

In Fig. 1, several examples of cortical breaks on corresponding HR-pQCT and μCT images are presented. Panel A and B show a cortical break on both HR-pQCT and μCT. In panel C, a discontinuity of the cortex is found on HR-pQCT. However, it did not meet the definition of a cortical break applied in this study, because it was visible on one slice only, leading to discrepancy with the results from μCT. In panel D, a cortical break was detected on HR-pQCT, but not on μCT, where a thin cortical lining was seen.

## Discussion

This study is the first that reports on aspects of reliability and validity of detecting cortical breaks in hand joints using HR-pQCT with μCT as gold standard. Cortical breaks were found in all joints with both imaging modalities. Intrareader reliability of HR-pQCT and μCT was moderate to substantial, while interreader reliability was fair to moderate. The sensitivity of HR-pQCT in detecting cortical breaks was high (81.6 %).

In our study, only cortical discontinuities meeting the definition of a break, ie clearly visible cortical interruptions on at least 2 HR-pQCT (or 9 μCT) consecutive slices in two planes, were scored as a cortical break. They may have different pathological or physiological backgrounds, such as erosions or vascular channels [8, 9]. A formal classification system for defining breaks visualized on HR-pQCT and μCT is lacking. Histological examination is needed to provide more insight in the nature of these cortical breaks and for developing definitions.

In a previous study by Stach et al. an almost perfect and substantial intra- and interreader reliability was reported using HR-pQCT for grading bone lesions and discrimination between healthy individuals and RA patients (k = 0.82 and k = 0.75 respectively), but reliability on the presence and number of cortical breaks was not reported [8]. The precision in scoring abnormalities visualized with several imaging techniques varies widely, even by experienced readers, as has been demonstrated for example for scoring radiographs in RA (ICC ranged from 0.65 to 0.99) [14]. In general, lower values for interreader reliability in comparison with intrareader reliability are reported [15], corresponding to our findings. In our study, the breaks were scored visually, which is reader dependent. An automatic scoring algorithm, with detection of pre-defined definitions of breaks and executed automatically by the computer, could potentially improve reliability by minimizing reader interventions.

We investigated the sensitivity of HR-pQCT in detecting cortical breaks with μCT as the gold standard and found a high sensitivity (81.6 %). Unfortunately, no comparative studies are available. In contrast, two studies used HR-pQCT as the reference method for investigating the sensitivity to detect cortical breaks of other imaging modalities [9, 16]. These studies reported a sensitivity of 85.7 % for MRI, 60.9 % for CR, and 83–100 % for ultrasound with HR-pQCT as the reference method [9, 16]. We found a lower specificity of HR-pQCT in detecting cortical breaks (64 %) in comparison to sensitivity. A possible explanation for this could be a phenomenon attributed to a partial volume effect leading to a reduced cortical signal on HR-pQCT, giving the impression that a cortical break is present, whereas on μCT the cortex is intact. An example of this is shown in Fig. 1, panel D.

There are several limitations of this study. First, we evaluated whether the total number of breaks counted per quadrant corresponded between the two imaging modalities, but did not consider correspondence in exactly the same location. This might have led to an overestimation of the reliability. Second, we used fingers from cadaver specimens with unknown medical history and a relatively high mean age (85.1 years). Due to the old age of the donors, and the preservation in formalin, the cortices might become less mineralized [17]. The average vBMD of the specimens was 245 mgHA/cm^3^, which is some 20 % lower than the average in the normal population (>300 mgHA/cm^3^) [10, 18]. This may hamper the scoring of a cortical break on HR-pQCT. It is also possible that thin regions were falsely identified as a cortical break. However, the use of cadaveric specimens was essential as in vivo human subjects cannot be measured by μCT because of a long scanning time. Third, the cadaveric specimens had slightly different orientations in the HR-pQCT versus the μCT scanner. Despite the careful visual matching of the regions of interest on HR-pQCT and μCT, the angle at which the transversal images were viewed was slightly different in some joints and a cortical break might therefore be missed. Fourth, a discrepancy between the readers regarding the number of cortical breaks identified on μCT was noticed. μCT images provide much detail, and in particular very small cortical interruptions were not always picked up by Reader 1. This indicates that, when visually analyzing μCT images, more stringent definitions are necessary than when using HR-pQCT because of the higher resolution.

## Conclusions

Cortical breaks were commonly visualized in hand joints with HR-pQCT and μCT. Reliability of both HR-pQCT and μCT was fair to moderate. HR-pQCT was sensitive to detect cortical breaks with μCT as gold standard. In spite of the limitations of our study, including the discrepancy of μCT results between the readers, we have shown that HR-pQCT is highly sensitive to detect cortical breaks with a fair to moderate reliability compared to μCT. Our findings need further evaluation, preferably with focus on histological analyses to clarify the nature of the breaks and to establish more reliable definitions and a classification system for analyzing cortical breaks on high-resolution CT images.

## Abbreviations

CR, conventional radiographs; CT, computed tomography; DICOM, digital imaging and communications in medicine; HR-pQCT, high-resolution peripheral quantitative computed tomography; ICC, intraclass correlation coefficient; MCP, metacarpophalangeal; PIP, proximal interphalangeal; PPV, positive predictive value; RA, rheumatoid arthritis; k, Cohen’s Kappa; μCT, microCT; μm, micrometer

## Additional files

Additional file 1: Method of selection of regions of interest. Method of selection of regions of interest in an MCP and PIP joint on HR-pQCT and μCT. Total scan area for an MCP joint on HR-pQCT was 18.04 mm and for a PIP joint 9.02 mm. Total scan area for an MCP joint on μCT was 15.26 mm and for a PIP joint 9.45 mm. Abbreviations: HR-pQCT; high-resolution peripheral quantitative computed tomography, μCT; micro computed tomography, MCP; metacarpophalangeal, PIP; proximal interphalangeal. (TIF 11020 kb) Additional file 2: Resolution of HR-pQCT and μCT imaging. Resolution of HR-pQCT imaging is 82 μm, while the resolution of μCT imaging is 18 μm. To match both resolutions, two consecutive slices on HR-pQCT correspond with 9 consecutive slices on μCT. Abbreviations: HR-pQCT; high-resolution peripheral quantitative computed tomography, μCT; microCT. (TIF 3952 kb) Additional file 3: Division of a phalangeal base of an MCP joint. Phalangeal base of an MCP joint divided into palmar, ulnar, dorsal and radial quadrants. Abbreviations: MCP; metacarpophalangeal (TIF 9718 kb) Additional file 4: Examples of μCT images. Examples of μCT images that could have attributed to the differences in scoring cortical breaks between Reader 1 and 2. Panel a. Large cortical breaks (*arrow*) show high agreement. Panel b. Small cortical breaks (*arrow*) show less agreement. Panel c. An extremely thin cortex (*arrow*). Panel d. One large break (*in brackets*) was counted by Reader 1, where Reader 2 considered this as several smaller cortical breaks (*arrow*). Abbreviations: μCT; microCT. (PNG 460 kb) Additional file 5: 2 × 2 contingency tables. 2 × 2 contingency tables for Reader 1 (all joints and MCP and PIP separately) and Reader 2 (all joints and MCP and PIP joints separately). (DOCX 17 kb)
