# Supplementary figures and images for: Visual detection of cortical breaks in hand joints: reliability and validity of high-resolution peripheral quantitative CT compared to microCT
Source: BMC Musculoskelet Disord. 2016 Jul 11;17:271. doi: 10.1186/s12891-016-1148-y (PMC4940720; doi:10.1186/s12891-016-1148-y)

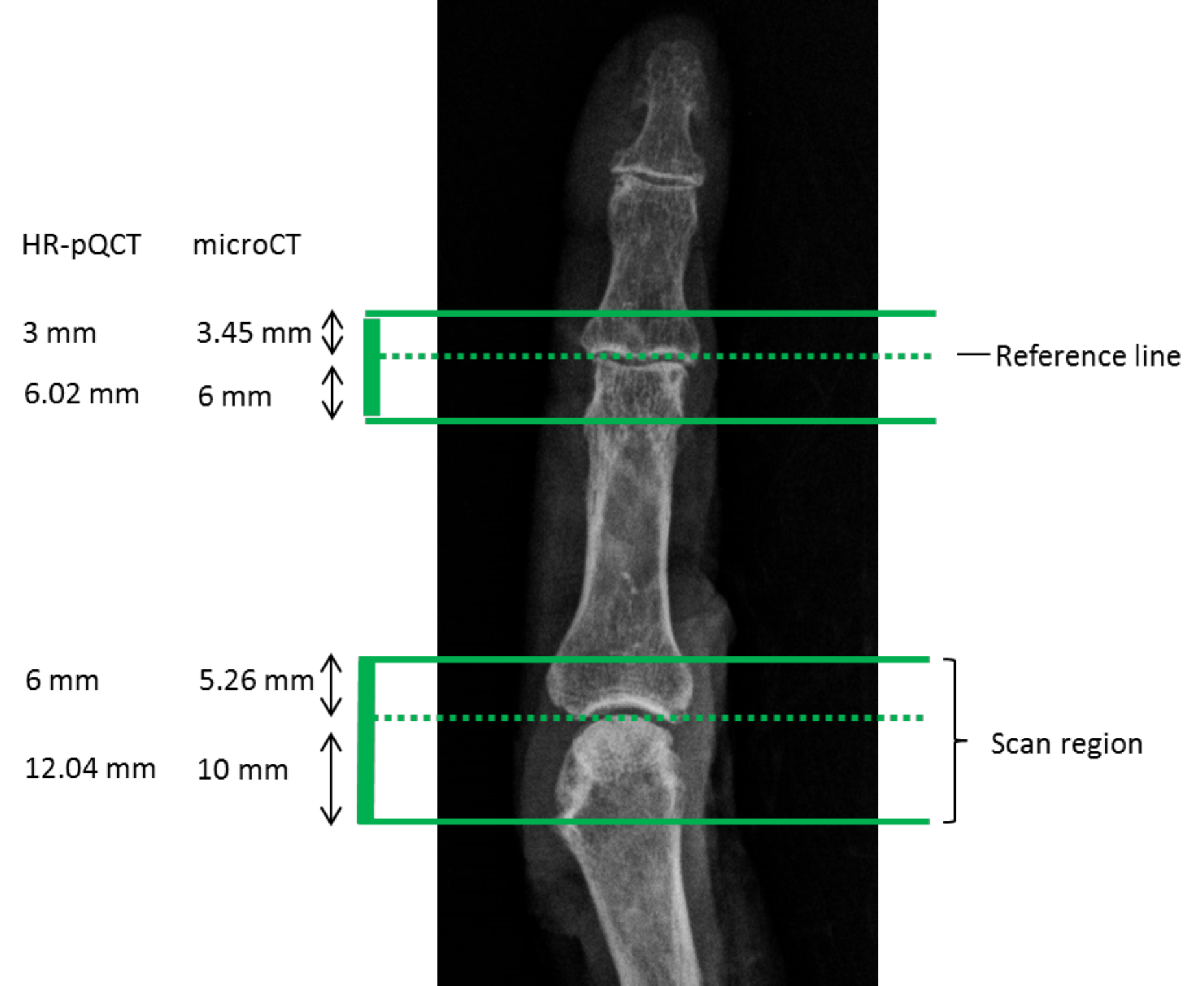

Supplement: Additional file 1: — Method of selection of regions of interest. Method of selection of regions of interest in an MCP and PIP joint on HR-pQCT and μCT. Total scan area for an MCP joint on HR-pQCT was 18.04 mm and for a PIP joint 9.02 mm. Total scan area for an MCP joint on μCT was 15.26 mm and for a PIP joint 9.45 mm. Abbreviations: HR-pQCT; high-resolution peripheral quantitative computed tomography, μCT; micro computed tomography, MCP; metacarpophalangeal, PIP; proximal interphalangeal. (TIF 11020 kb) [file 12891_2016_1148_MOESM1_ESM.tif]

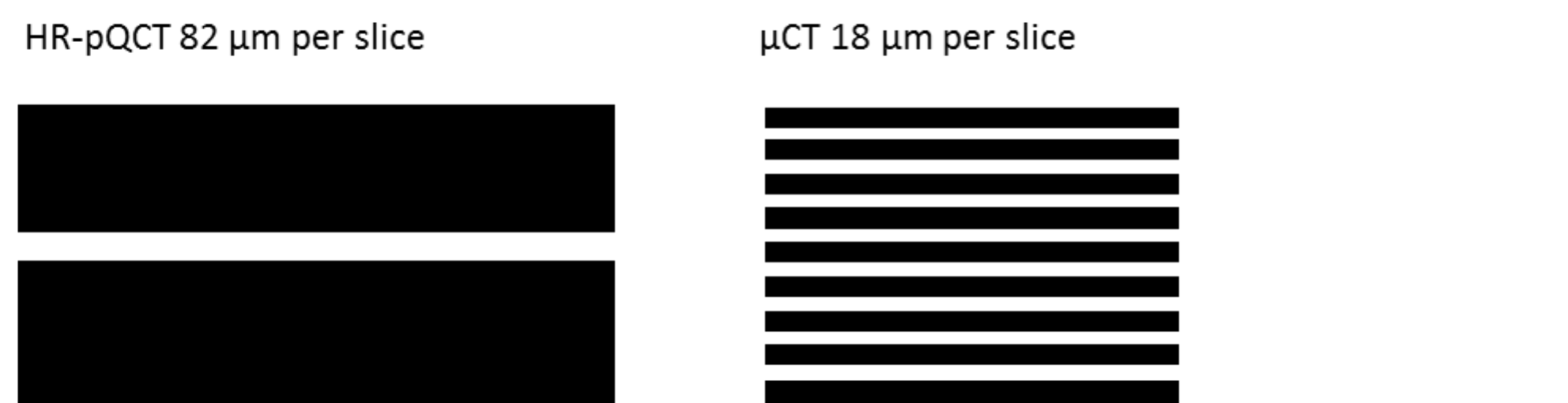

Supplement: Additional file 2: — Resolution of HR-pQCT and μCT imaging. Resolution of HR-pQCT imaging is 82 μm, while the resolution of μCT imaging is 18 μm. To match both resolutions, two consecutive slices on HR-pQCT correspond with 9 consecutive slices on μCT. Abbreviations: HR-pQCT; high-resolution peripheral quantitative computed tomography, μCT; microCT. (TIF 3952 kb) [file 12891_2016_1148_MOESM2_ESM.tif]

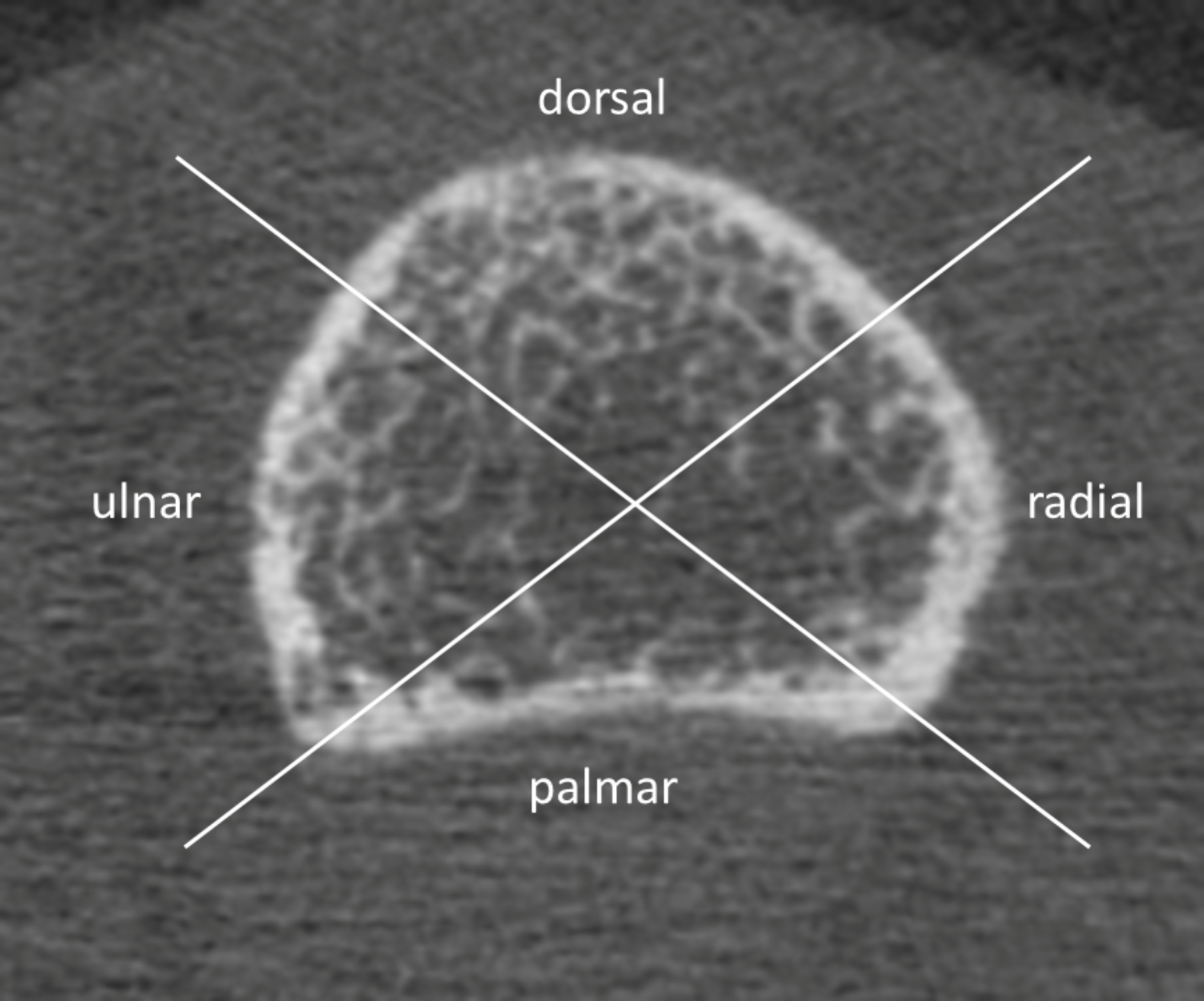

Supplement: Additional file 3: — Division of a phalangeal base of an MCP joint. Phalangeal base of an MCP joint divided into palmar, ulnar, dorsal and radial quadrants. Abbreviations: MCP; metacarpophalangeal (TIF 9718 kb) [file 12891_2016_1148_MOESM3_ESM.tif]

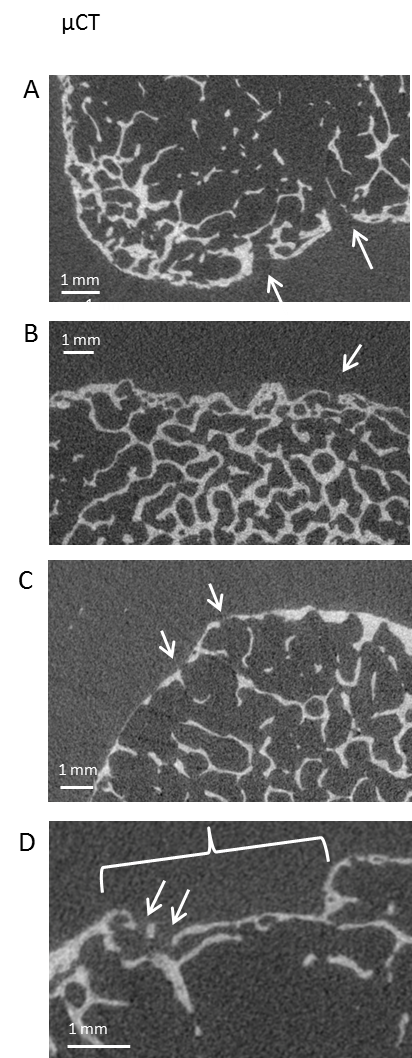

Supplement: Additional file 4: — Examples of μCT images. Examples of μCT images that could have attributed to the differences in scoring cortical breaks between Reader 1 and 2. Panel a. Large cortical breaks (arrow) show high agreement. Panel b. Small cortical breaks (arrow) show less agreement. Panel c. An extremely thin cortex (arrow). Panel d. One large break (in brackets) was counted by Reader 1, where Reader 2 considered this as several smaller cortical breaks (arrow). Abbreviations: μCT; microCT. (PNG 460 kb) [file 12891_2016_1148_MOESM4_ESM.png]
