# Supplementary material for: Visual detection of cortical breaks in hand joints: reliability and validity of high-resolution peripheral quantitative CT compared to microCT
Source: BMC Musculoskelet Disord. 2016 Jul 11;17:271. doi: 10.1186/s12891-016-1148-y (PMC4940720; doi:10.1186/s12891-016-1148-y)
Supplement: Additional file 5: — 2 × 2 contingency tables. 2 × 2 contingency tables for Reader 1 (all joints and MCP and PIP separately) and Reader 2 (all joints and MCP and PIP joints separately). (DOCX 17 kb) [file 12891_2016_1148_MOESM5_ESM.docx]

| Table 1. 2x2 contingency table for Reader 1 all joints (MCP and PIP) | | | |
| --- | --- | --- | --- |
|  | uCT presence break | uCT absence break | Total |
| HR-pQCT presence break | 80 | 18 | 98 |
| HR-pQCT absence break | 18 | 32 | 50 |
| Total | 98 | 50 | 148 quadrants |

2x2 table on the presence of a cortical break on μCT (gold standard) and HR-pQCT of Reader 1 for all joints (MCP and PIP). Reader 1 considered 4 quadrants not evaluable due to the quality of the images due to a protocol error.

| Table 2. 2x2 contingency table for Reader 1 for MCP joints | | | |
| --- | --- | --- | --- |
|  | uCT presence break | uCT absence break | Total |
| HR-pQCT presence break | 46 | 7 | 53 |
| HR-pQCT absence break | 10 | 13 | 23 |
| Total | 56 | 20 | 76 quadrants |

2x2 table on the presence of a cortical break on μCT (gold standard) and HR-pQCT of Reader 1 for MCP joints. Reader 1 considered 4 quadrants not evaluable due to the quality of the images due to a protocol error.

| Table 3. 2x2 contingency table for Reader 1 for PIP joints | | | |
| --- | --- | --- | --- |
|  | uCT presence break | uCT absence break | Total |
| HR-pQCT presence break | 34 | 11 | 45 |
| HR-pQCT absence break | 8 | 19 | 27 |
| Total | 42 | 30 | 72 quadrants |

2x2 table on the presence of a cortical break on μCT (gold standard) and HR-pQCT of Reader 1 for PIP joints. Reader 1 considered 4 quadrants not evaluable due to the quality of the images due to a protocol error.

| Table 4. 2x2 contingency table for Reader 2 for all joints (MCP and PIP joints) | | | |
| --- | --- | --- | --- |
|  | uCT presence break | uCT absence break | Total |
| HR-pQCT presence break | 71 | 15 | 86 |
| HR-pQCT absence break | 32 | 34 | 66 |
| Total | 103 | 49 | 152 quadrants |

2x2 table on the presence of a cortical break on μCT (gold standard) and HR-pQCT of Reader 2 for all joints (MCP and PIP).

| Table 5. 2x2 contingency table for Reader 2 for MCP joints | | | |
| --- | --- | --- | --- |
|  | uCT presence break | uCT absence break | Total |
| HR-pQCT presence break | 42 | 6 | 48 |
| HR-pQCT absence break | 22 | 10 | 32 |
| Total | 64 | 16 | 80 quadrants |

2x2 table on the presence of a cortical break on μCT (gold standard) and HR-pQCT of Reader 2 for MCP joints.

| Table 6. 2x2 contingency table for Reader 2 for PIP joints | | | |
| --- | --- | --- | --- |
|  | uCT presence break | uCT absence break | Total |
| HR-pQCT presence break | 31 | 7 | 38 |
| HR-pQCT absence break | 10 | 24 | 34 |
| Total | 41 | 31 | 72 quadrants |

2x2 table on the presence of a cortical break on μCT (gold standard) and HR-pQCT of Reader 2 for PIP joints.
